# Assessing serum levels of SM22α as a new biomarker for patients with aortic aneurysm/dissection

**DOI:** 10.1371/journal.pone.0264942

**Published:** 2022-03-31

**Authors:** Ning Zhang, Ying-Ying Wang, Hai-Juan Hu, Gang Lu, Xin Xu, Yong-Qing Dou, Wei Cui, She-Jun Gao, Mei Han

**Affiliations:** 1 Key Laboratory of Medical Biotechnology of Hebei Province, Department of Biochemistry and Molecular Biology, College of Basic Medicine, Cardiovascular Medical Science Center, Hebei Medical University, Shijiazhuang, China; 2 Department of Functional Region of Diagnosis, The Fourth Affiliated Hospital, Hebei Medical University, Shijiazhuang, China; 3 Department of Cardiovascular Medicine, The Second Affiliated Hospital, Hebei Medical University, Shijiazhuang, China; 4 Department of Clinical Laboratory, The Fourth Affiliated Hospital, Hebei Medical University, Shijiazhuang, China; 5 Key Laboratory of Integrative Medicine on Liver-kidney patterns of Hebei Province, College of Integrated Chinese and Western Medicine, Hebei University of Chinese Medicine, Shijiazhuang, China; Stellenbosch University Faculty of Medicine and Health Sciences, SOUTH AFRICA

## Abstract

**Background:**

Aortic aneurysm/dissection (AAD) is now encountered more often because of the increasing prevalence of atherosclerosis and hypertension in the population. Despite many therapeutic improvements, in particular timely and successful surgery, in-hospital mortality rates are still higher. Timely identification of patients at high risk will help improve the overall prognosis of AAD. Since early clinical and radiological signs are nonspecific, there is an urgent need for accurate biomarkers. Smooth muscle 22α (SM22α) is a potential marker for AAD because of its abundant expression in vascular smooth muscle, which is involved in development of AAD.

**Methods:**

We prepared three different mouse models, including abdominal aortic aneurysm, neointimal hyperplasia and atherosclerosis. SM22α levels were assessed in serum and vascular tissue of the mice. Next, the relationships between serum SM22α level and vascular lesion were studied in mice. Finally, serum from 41 patients with AAD, 107 carotid artery stenosis (CAS) patients and 40 healthy volunteers were tested for SM22α. Serum levels of SM22α were measured using an enzyme-linked immunosorbent assay (ELISA).

**Results:**

Compared with the controls, serum SM22α levels were reduced in the models of aortic aneurysm, neointimal formation and atherosclerosis, and elevated in mice with ruptured aneurysm. Serum SM22α level was negatively correlated with apoptosis rate of vascular smooth muscle cells (VSMC), ratio of intima/ media (I/M) area and plaque size. Patients with AAD had significantly higher serum SM22α levels than patients with only CAS, or normal controls.

**Conclusion:**

Serum SM22α could be a potential predictive marker for AAD, and regulation of VSMC is a possible mechanism for the effects of SM22α.

## Introduction

Aortic aneurysm/dissection (AAD) is one of the most life-threatening disease, associated with high rates of mortality in case of aortic rupture [1,2]. For all patients with AAD, the early diagnosis and treatment is crucial for improved survival. However, due to the lack of typical clinical symptoms, AAD is often misdiagnosed as angina, acute myocardial infarction or other cardiovascular diseases, resulting in the delay of treatment. Currently, the diagnosis and follow-up of AAD mainly depends on various imaging techniques, such as magnetic resonance angiography, computed tomography angiography, or ultrasound, which are relatively time-consuming in some emergency situations. Therefore, blood-derived biomarkers may be more suitable for the rapid diagnosis of AAD.

The structural and functional changes in the vascular smooth muscle cells (VSMC) including phenotypic transformation and apoptosis play a critical role in the pathogenesis of vascular remodeling. Loss of VSMC functions and activation of inflammation are thought to weaken the arterial wall and increase the risk of AAD formation and rupture [3–7].

Smooth muscle 22α (SM22α) is an actin-associated protein abundant in contractile VSMC and is widely used as a phenotypic marker to identify VSMC phenotypic transformation. SM22α decorates the contractile filament bundles within cultured VSMC exhibiting differentiated phenotypes. Decrease in expression of SM22α has been demonstrated in human atherosclerotic lesions, neointima formation [8,9], abdominal aortic aneurysm (AAA) [10,11], and thoracic aortic dissection [12], and mouse models [13,14]. Our previous studies demonstrate that the arteries of *Sm22α*^-/-^ mice develop enhanced inflammatory response and ROS production, which is involved in aortic aneurysm through different signaling mechanisms [15–18], suggesting that SM22α expression is closely associated with the structure and function of vascular media.

However, a report directly addressing circulating SM22α levels in AAD patients is still lacking. Thus, on the basis of such premises, we investigated serum SM22α level in patients with AAD compared with patients with only carotid artery stenosis (CAS) and normal controls, and assessed it value as diagnostic and monitoring markers in AAD.

## Materials and methods

### Animals

Male C57BL/6J mice were obtained at 6~8 weeks of age from the Hebei Medical University Experimental Animal Center (Hebei, China). Mice were maintained under standard conditions at 22°C with a 12-hour light/dark cycle and free access to food and water. For the induction of AAA, C57BL/6J mice were induced by perivascular application of CaPO_4_. A small piece of gauze soaked in 0.5M CaCl_2_ was applied perivascularly for 15 min. This gauze is then replaced with another piece of phosphate buffered saline (PBS)-soaked gauze for 5 min. Aortic aneurysm rupture model of mice was infused with β- aminopropionitrile (BAPN 300 mg/kg/d) combined with Ang II (4.0 mg/kg/d) simultaneously for 21 days via intraperitoneal injection. BAPN and Ang II were purchased from Sigma (St. Louis, MO). The neointimal formation of the C57BL/6J mice was induced by complete ligation of the left common carotid artery for 14~28 days. *Ldlr*^−/−^ mice (at 6~8 weeks of age from Ex&Invivo Biotech Co.td, Hebei, China) were fed a high fat diet (HFD) containing 0.5% cholesterol and 20% fat for 12 weeks to induce atherosclerosis development. All animals were euthanized using intraperitoneal overdose anesthesia with sodium pentobarbital (200~250 mg/kg) at the end of the experiments, and the vascular tissues and blood samples were collected. All animal procedures conformed to the Guide for the Care and Use of Laboratory Animals published by the US National Institutes of Health (NIH Publication, 8th Edition, 2011), and were approved by the Institutional Animal Care and Use Committee of Hebei Medical University.

### Patients

The Medical Ethics Committee of Hebei Medical University approved all protocols using human samples (No. 2017042). All participants provided written informed consent prior to their participation in the study.

From May 2020 to March 2021, according to the computed tomography angiography results, clinical features, and diagnostic clinical results, a total of 41 AAD patients were admitted to the Vascular Surgery Department of the Fourth Affiliated Hospital of Hebei Medical University for their first-ever AAD. All of these people were treated via emergency surgical repair. May 2020 was chosen at the commencement time for this study and patients were selected on the basis of the following inclusion criteria: (1) emergency surgical treatment of Stanford type A dissection or type B dissection thoracic aneurysm; (2) no history of neoplasm or autoimmune, infectious, or inflammatory systemic diseases; (3) no presence of genetic syndromes known to be responsible for aortic disease. Serum samples were collected at 5 time points: preoperative, day 1, 5, 7 and 14 postoperative.

107 CAS patients were included to assess the difference in serum SM22α between AAD and atherosclerotic carotid stenosis disease in the same period. All patients were from the Department of Cardiovascular Medicine and underwent Duplex Carotid Ultrasound examination, which were enrolled on the basis of the following inclusion criteria: (1) when a plaque was identified in the carotid artery, the following degree of stenosis categorized by hemodynamic criteria were recorded: mild (<50%), moderate (50%~69%), and severe stenosis (≥70%); (2) no cardiac causes of stroke; (3) no history of neoplasm or autoimmune or inflammatory systemic diseases; and (4) no familiar or personal history of AAD.

40 normal controls (NC group) matched for age and sex were included in this study to obtain reference values for serum levels of SM22α. These were selected on the basis of the following exclusion criteria: (1) no presence of genetic syndromes known to cause aortic disease; (2) no family history of AAD or atherosclerotic cardiovascular disease; (3) no history of AAD or atherosclerotic disease; (4) no diabetes mellitus; (5) no dyslipidaemia; and (6) no uncontrolled hypertension.

Since this was a retrospective observational study, the sample size was informed by the available participants during the study period rather than as a result of a prespecified sample size estimate.

### Study variables and criteria

Clinical data including patient age, sex, smoking, drinking and blood pressure were recorded during clinical reviews or were obtained from previous hospital admission records. Smoking was defined as current smoking (smoking within the last month). Drinking was defined as an intake of more than one standard cup of Chinese liquor, one large bottle of regular beer, or one double measure of red wine at a time more than three times a week. Blood pressure was measured from each patient’s upper right arm in a sedentary position using an automated sphygmomanometer after a 5-min rest. Hypertension was defined as an office blood pressure of 140/90 mmHg and above. All blood variables, including levels of fasting plasma glucose (FPG), serum total cholesterol (TC), triglyceride (TG), high-density lipoprotein cholesterol (HDL-c) and low- density lipoprotein cholesterol (LDL-c), were measured concomitantly. Abnormal blood glucose was diagnosed at FPG≥7.0 mmol/L. Dyslipidemia was generally defined as the serum level of TC of 6.19 mmol/L and above, the serum level of TG of 2.27 mmol/L and above, the serum level of LDL-c of 4.14 mmol/L and above.

### Measurement of SM22α level in serum

Serum levels of SM22α were measured using enzyme-linked immunosorbent assay (ELISA), according to the manufacturer’s instructions (cat.EK6896 & cat.EK6897, Signalway Antibody, USA). All serum samples should be tested initially without any dilution. Final SM22α serum levels were obtained based on the dilution of samples which corresponded to the linear portion of the standard curve.

### Immunofluorescence analysis

Immunofluorescence staining was performed on 6-μm-thick frozen sections. Sections were blocked using 5% normal goat serum in TBS for 30 min and then incubated with primary antibodies against SM22α (Abcam, ab14106, 1:100) at 4°C overnight, and isotype matched controls. Sections were washed 3 times with TBS and incubated with fluorescein-conjugated secondary antibodies (Alexa Fluor^®^543, Invitrogen) at a 1/200 dilution for 1 h at room temperature. Nuclei were detected by DAPI (Antifade Mountant with DAPI, Thermofisher). Images were acquired using a Confocal Laser Scanning Microscope Systems (Leica). Digitized images were analyzed with software program LAS AF Lite.

### Western blotting

RIPA buffer (50 mM Tris-HCl, pH 7.5, 1% NP-40, 0.5% Na-deoxycholate, 0.1% SDS, 1 mM EDTA, 150 mM NaCl supplemented with complete proteinase inhibitor) was used to extract the whole protein from the neointimal hyperplasia, AAA and atherosclerotic tissues of mice. To ensure the same amount of proteins for each sample, supernatant was quantified by Bradford protein assay. Equal amounts of protein (30~60 μg) were separated by 10% SDS-PAGE, and electro-transferred to a PVDF membrane. Membranes were blocked with 5% non-fat milk in tris-buffered saline-Tween for 1 h at room temperature, and incubated with primary antibodies against SM22α (Abcam, ab14106, 1:1000), α-SMA (Abcam, ab5694, 1:1000), OPN (Proteintech, 22952-1-AP, 1:500) and β-actin (Santa Cruz, sc-47778, 1:1000) at 4°C overnight. The membranes were then incubated with horseradish peroxidase-conjugated anti-mouse IgG (Abcam, ab205719, 1:20000) or anti-rabbit IgG (Abcam, ab205718, 1:20000) for 1 h at room temperature. The blots were evaluated with GE Image Quant^™^ LAS 4000 detection system. The protein bands of interest were quantified using Image Pro Plus 6.0 software, and the integrated signal densities were normalized to β-actin (the loading control).

### RNA isolation and quantitative reverse transcription-PCR (qRT -PCR)

Total RNAs from vascular tissues of mice were isolated using TRIzol reagent (Life Technologies). The nuclear and cytoplasmic fractions were extracted using Minute^™^ Cytoplasmic and Nuclear Extraction Kit (Invent Bio technologies). To quantify the amount of mRNA and circRNA, cDNAs were synthesized using the M-MLV First Strand Kit (Life Technologies), and quantitative PCRs were performed using SYBR Green qPCR SuperMix-UDG (Life Technologies). For quantification, all RNA expression was normalized to the amount of Tubulin using the 2^-ΔΔCt^ method. For RT-PCR analysis, the following specific primers were used: *SM22α* forward, 5΄-CAACAAGGGTC CATCCTACGG-3΄ and reverse, 5΄-ATCTGGGCGGCCTACATCA-3΄; *β-actin* forward, 5΄-CGAGGCCCAGAGCAAGAGAGGTAT-3΄ and reverse, 5΄-CAC GGTTGGCCTTAGGGTTCA-3΄.

### Statistics

Data analysis was performed with SPSS version 16.0. Data are presented as the means ± standard error of the mean (SEM), while categorical variables are presented as numbers or percentages. Differences between two groups were compared by *t*-tests. Associations were analyzed using Pearson correlations and linear regression models. The clinically important variables were selected for multiple linear regression analysis. Receiver operating characteristic (ROC) curve analysis was performed to assess the area under the curve (AUC) and Youden index was used to determine the best cut-off value of serum SM22α levels for predicting AAD in study subjects. Significance was taken at *P*<0.05 throughout, and denoted with one, two and three asterisks when lower than 0.05, 0.01 and 0.001, respectively.

## Results

### Serum SM22α levels are correlated with vascular disease in mice

**Fig 1:**
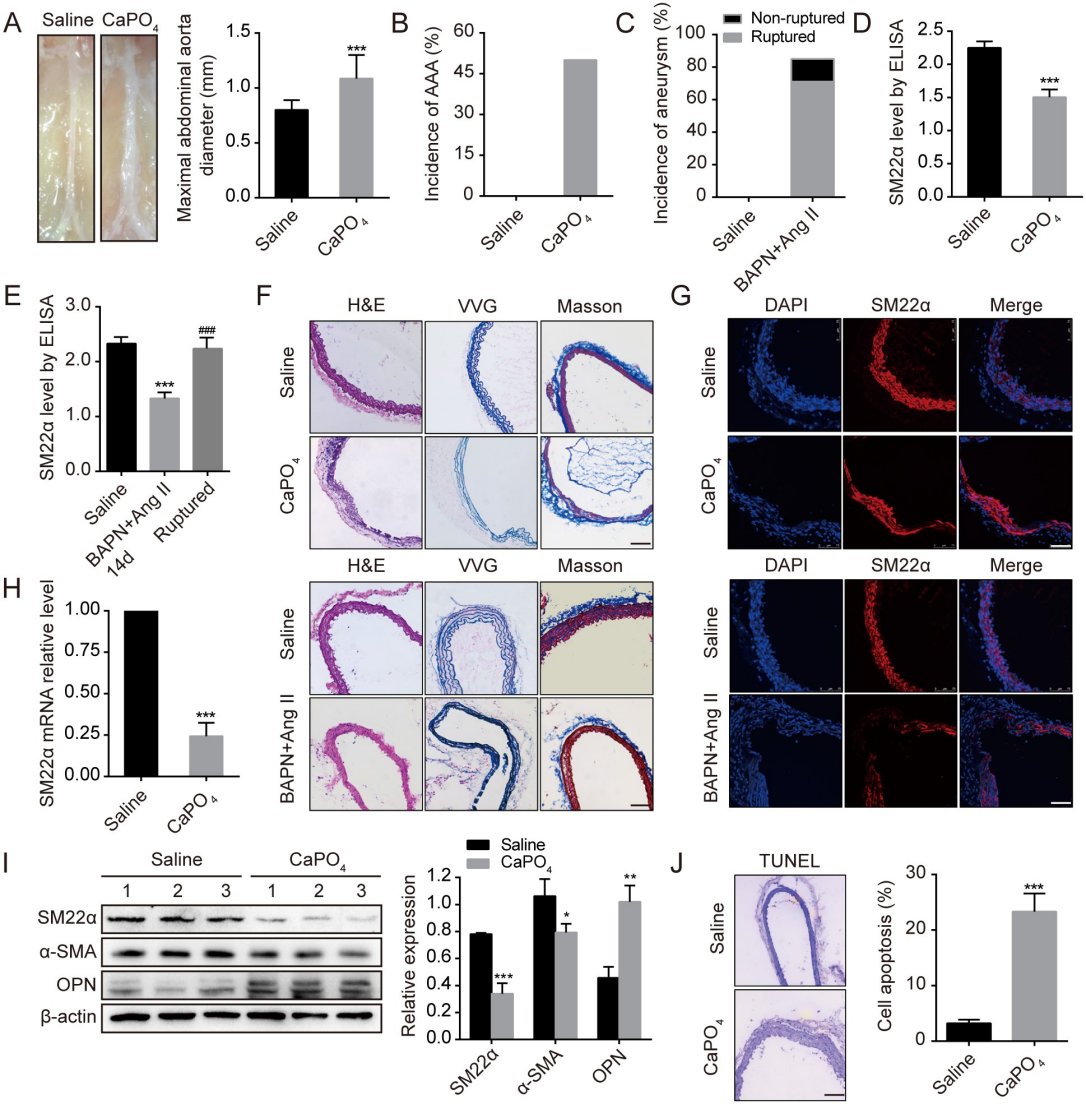
SM22α expression of serum samples and tissues in mice subjected to aneurysm compared with their histological perturbations. (A-B) The maximal abdominal aortic diameter and the incidence of AAA in Saline or CaPO_4_-induced group (n = 8). (C) The incidence of aneurysm rupture in WT mice (n = 7) after BAPN and Ang II administration for 3 weeks. (D) SM22α serum levels from CaPO_4_ induced WT mice compared with Saline (n = 8). (E) SM22α serum levels from BAPN and Ang II induced WT mice (Saline and BAPN+Ang II 14d group, n = 7 per group, Ruptured group, n = 5). (F-G) H&E, VVG, Masson and immunofluorescence staining for SM22α were arranged each for aortic aneurysm tissues and control aortic tissues (n = 5). Scale bars, 100μm. (H-I) The expression of SM22α mRNA and protein in aortic aneurysm tissues and control aortic tissues (n = 3). (J) The apoptosis rate of VSMC was detected by TUNEL in Saline or CaPO_4_-induced group (n = 6) Scale bars, 100μm. All data are expressed as mean±SEM. *P<0.05, **P<0.01, ***P<0.001 vs controls; ^###^*P*<0.001 vs BAPN+Ang II 14d. **WT**, wild-type; **BAPN**, β-aminopropionitrile; **Ang II**, angiotensin II.

We showed that the incidence of AAA induced by CaPO_4_ was 50% (4/8). Obviously, the maximum diameter of abdominal aorta was enlarged in mice induced by CaPO_4_ (*P*<0.001, Fig 1A and 1B), accompanied by increased elastin disruption and degradation (Fig 1F). Immunofluorescence staining, qRT-PCR and Western blotting indicated that the expression of SM22α was remarkably decreased in AAA tissues compared with normal aortic tissues (*P*<0.001), and negatively correlated with the maximum diameter of abdominal aorta (*r* = -0.825, *P*<0.05, Fig 1G–1I). To validate VSMC phenotypic states in this study, we also detected other markers of phenotypic switching, including contractile VSMC markers (α-SMA) and synthetic VSMC marker (OPN). Expression of SM22α and α-SMA were significantly decreased while the expression of OPN was significantly increased in aortic aneurysm tissues compared to control aortic tissues. The phenotypic changes of VSMC are from constriction to synthesis, which can lead to change of tunica media character and eventually lead to aortic aneurysm.

Aortic aneurysm rupture model of mice was established by administering β-aminopropionitrile (BAPN), a lysyl oxidase inhibitor, and angiotensin II (Ang II) to induce hypertension and degeneration of the elastic lamina, which would eventually result in the onset of aneurysm rupture. In this model, cotreatment with BAPN and Ang II caused an 85.7% (6/7) aneurysm incidence, with 71.4% (5/7) of these having aortic aneurysm rupture (Fig 1C).

Similarly, histomorphological and immunofluorescence staining showed that the expression of SM22α in aneurysm tissues was significantly decreased with the development of aneurysm (Fig 1G).

As shown in Fig 1D and 1E, serum SM22α levels were decreased in AAA model of mice induced with CaPO_4_ (*P*<0.001), which were consistent with reduction of SM22α levels in lesion tissues and negatively correlated with the rate of smooth muscle cells apoptosis in arterial media (*r* = -0.778, *P*<0.05, Fig 1J). Similar changes were found in a mouse model of BAPN and Ang II-induced aortic aneurysm formation and rupture. At an early stage of aneurysm development (after 2 weeks of co-administration of BAPN and Ang II), serum SM22α levels were markedly decreased compared with saline group (*P*<0.001), and significantly elevated in mice with ruptured aneurysm after 3 weeks administration (*P*<0.001), suggesting that it may be associated with SM22α released into the blood stream during the rupture process of aneurysm.

**Fig 2:**
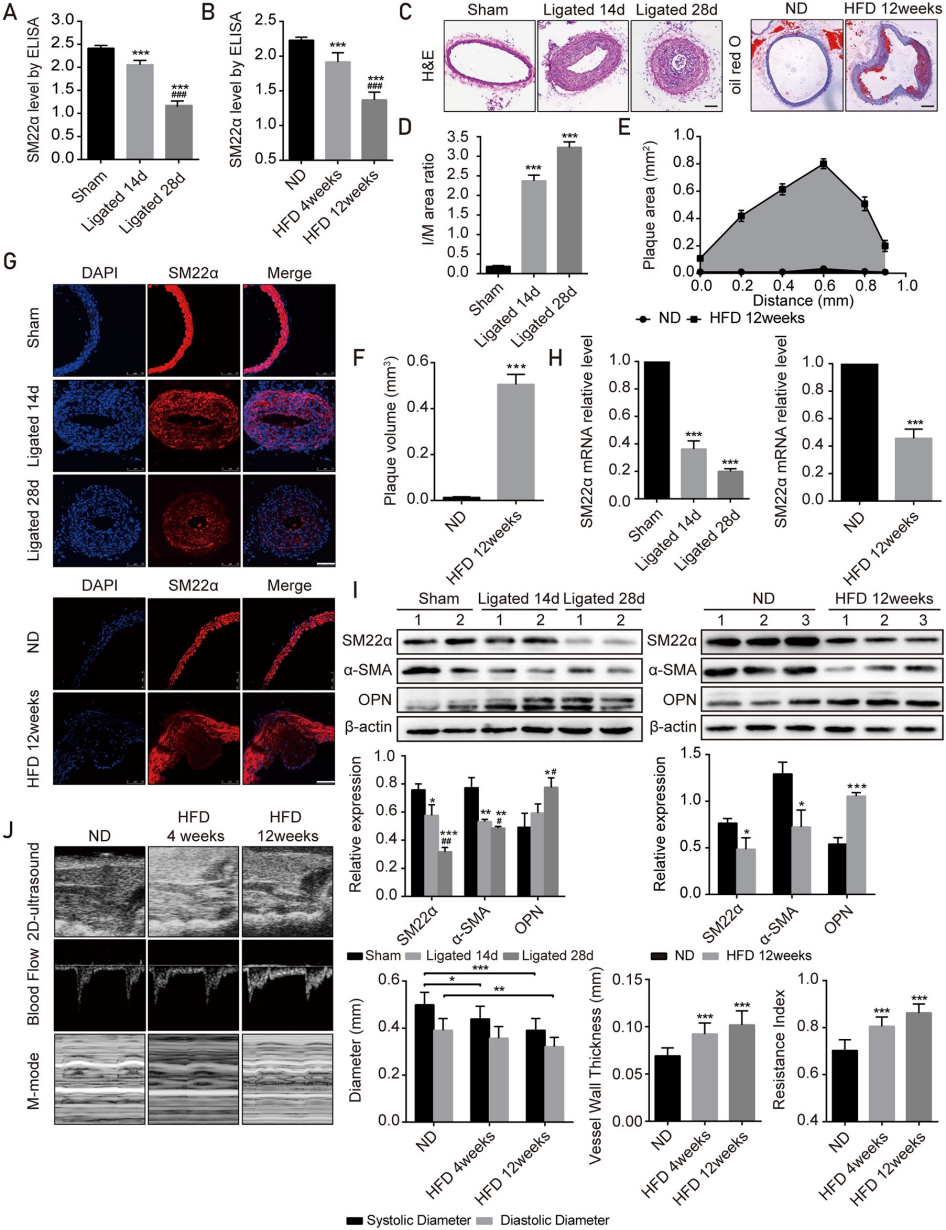
Serum SM22α level in models of common carotid artery ligation and HFD-fed mice is paralleled with its expression in vascular tissues. (A-B) SM22α levels of serum samples in models of carotid artery ligation (n = 8) and HFD-fed mice (n = 10). (C) Representative images of H&E and oil red O-stained aorta. Scale bars, 250 μm. (D) The ratio of intimal/medial (I/M) area in Saline and carotid artery ligation mice aortas (n = 5). (E) Histologic quantification of plaque area at set distances from the aorta in HFD-fed mice (n = 6). (F) Lesion volume was calculated as area under the curve in (E). (G) Immunofluorescence staining for SM22α in each group. Scale bars, 100 μm. (H) Relative mRNA expression of SM22α in mice aortas of each group (n = 3). Gene expression was normalized to β-actin. (I) Western blot for the expression of SM22α in mice aortas of each group (n = 3). β-actin served as a loading control. (J) Carotid ultrasonic examination in normal diet (ND) and HFD group, including representative images, systolic diameter, diastolic diameter, vessel wall thickness, resistance index. All data are expressed as mean±SEM. *P<0.05, **P<0.01, ***P<0.001 vs controls; ^#^*P*<0.05, ^##^*P*<0.01, ^###^*P*<0.001 vs Ligated 14d or HFD 4weeks.

Expression of SM22α in tissues and serum specimens displays a similar profile in mice, which is also reflected in other arterial injury models in mice. During the neointimal formation induced by the carotid artery ligation, the expression of SM22α in the carotid artery ligation group was significantly decreased (Fig 2G–2I), accompanied with increased intima/media (I/M) area ratio (Fig 2C and 2D). SM22α expression was negatively correlated with I/M area ratio (*r* = -0.913, *P*<0.05). Similarly, the expression of SM22α in the athero- sclerotic plaque of HFD-fed mice was significantly decreased and negatively correlated with plaque size (*r* = -0.913, *P*<0.05, Fig 2E and 2F), suggesting that the expression of SM22α may reflect the structure and function of vascular media. A significant decrease in the serum levels of SM22α was observed in carotid artery ligation and HFD-fed models of mice compared with normal controls (*P*<0.001, Fig 2A and 2B), and decreased serum SM22α levels were associated with progression of vascular lesions (*P*<0.001).

We further explored whether the serum SM22α levels could be used to evaluate the severity of vascular media injury. The results showed that serum SM22α levels were negatively correlated with the I/M area ratio in neointimal formation (*r* = -0.874, *P*<0.001, Fig 2D) and plaque size of HFD-fed mice (*r* = -0.855, *P*<0.05, Fig 2E and 2F). Next, the carotid artery ultrasound results of HFD-fed mice showed that the systolic diameter and diastolic diameter were significantly decreased, the vessel wall thickness and the resistance index were significantly increased (Fig 2J). Correlation analysis showed that serum SM22α levels were negatively correlated with the vessel wall thickness and resistance index (*r* = -0.8745, *r* = -0.8714, *P*<0.001). These data indicated that SM22α serum levels closely paralleled the increasing degree of arterial injury and could be a potential serum marker for detecting the occurrence and development of arterial injury.

### Serum level of SM22α in patients with AAD and CAS

Serum SM22α levels were investigated in 41 patients diagnosed with AAD undergoing surgery and compared with 107 CAS patients and 40 normal controls (Table 1).

**Table 1:** Characteristics of the study participants.

Variable	NC	AAD	CAS
Gender (men/women)	21/19	32/9	80/27
Age (years)	53.43±10.06	54.29±12.33	66.26±11.25
Smoking [n (%)]	5 (12.50%)	7 (20.59%)	21 (19.63%)
Drinking [n (%)]	5 (12.50%)	8 (23.53%)	17 (15.89%)
SBP (mmHg)	127.70±7.47	153.82±9.41***	137.33±17.68
DBP (mmHg)	74.63±9.47	82.53±9.23	78.03±11.76
FPG (mmol/L)	5.10±0.93	4.77±0.81	5.27±2.27
TG (mmol/L)	1.54±0.85	3.29±0.61***	2.34±1.38***
TC (mmol/L)	4.87±0.80	3.24±1.00	4.03±1.60
HDL-c (mmol/L)	1.38±0.35	1.33±0.56	1.39±0.71
LDL-c (mmol/L)	3.13±0.64	2.73±1.04	2.82±1.40

Data are expressed as mean±SD, median (interquartile range), or n (%).

***P<0.001 vs NC group.

SBP, systolic blood pressure; DBP, diastolic blood pressure; FPG, fasting plasma glucose; TC, total cholesterol; TG, triglyceride; HDL-c, high-density lipoprotein cholesterol; LDL-c, low-density lipoprotein cholesterol.

**Fig 3:**
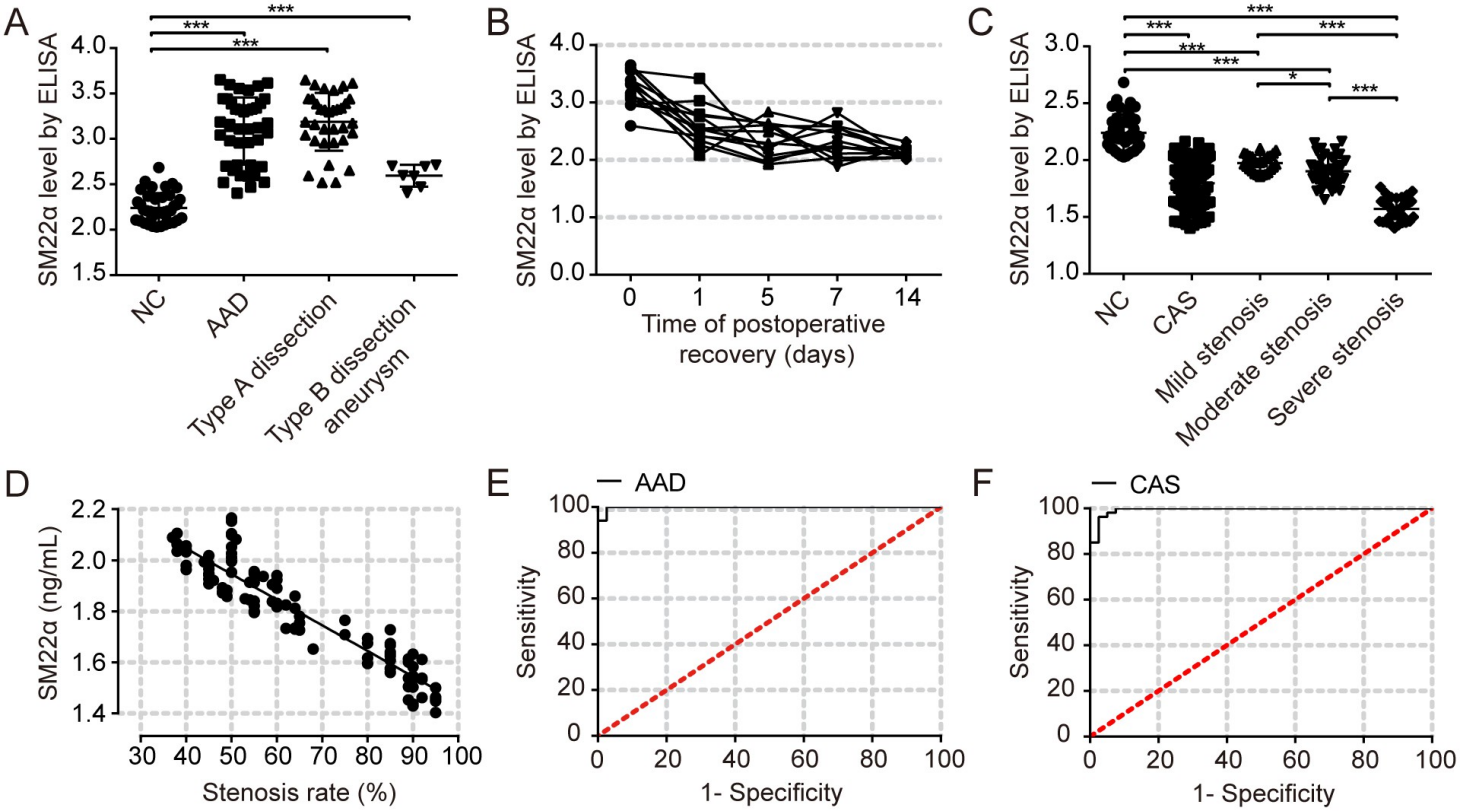
SM22α levels of serum samples in human. (A) The level of SM22α was assayed using an ELISA in human serum samples from normal controls (NC) group (n = 40), AAD patients (n = 41), type A dissection patients (n = 34), type B dissection aneurysm patients (n = 7). (B) Serum level of SM22α in the type A dissection patients (n = 13) at different days after arrival in the hospital. (C) Serum SM22α levels were significantly decreased in patients with CAS (n = 107) and comparison of serum SM22α in patients with different severities of stenosis. (D) Correlation of serum SM22α level with the degree of carotid stenosis in patients (*r* = -0.939, *P*<0.001). (E-F) The ROC curve analysis: The diagnostic prediction of serum SM22α for AAD and CAS. The AUC value was 0.996 and 0.977, respectively. AUC indicates area under the receiver operating curve. All data are expressed as mean±SEM. *P<0.01, **P<0.01 and ***P<0.001.

In this study, we simultaneously detected serum SM22α levels in patients with AAD before and after surgical intervention. As shown in Fig 3A and 3B, the serum SM22α level of AAD patients (n = 41) at admission (3.080±0.370 ng/mL) was significantly higher than that of the normal controls (2.297±0.122 ng/mL, *P*<0.001). Next, we compared the serum SM22α levels between type A dissection patients (n = 34) and type B dissection thoracic aneurysm patients (n = 7), serum SM22α levels were significantly increased in patients with type A dissection and type B dissection thoracic aneurysm (3.189±0.300 ng/mL, 2.595±0.121 ng/mL, respectively, *P*<0.05) compared with the normal controls. More interestingly, we observed sharp differences in serum SM22α levels between these two groups (*P*<0.05), speculating that the serum SM22α levels may be related to the lacerated range and degree. Serum SM22α levels were markedly decreased at day 1 after surgery, and it was significantly reduced to the normal range on the 14th day after operation (2.190±0.230 ng/mL, *P*<0.05).

In addition, we measured serum SM22α levels in 107 patients with CAS. These patients were categorized into mild (n = 27), moderate (n = 40) and severe (n = 40) carotid stenosis groups by ultrasonography, we found that serum SM22α levels were dramatically lower in CAS patients (1.797±0.204 ng/mL, Fig 3C). Meanwhile, in the patients with CAS, the levels of serum SM22α also showed significant differences with increasing stenosis. The reduction of serum SM22α level was the most obvious in severe stenosis group (1.572±0.091 ng/mL) compared with that in mild stenosis group and moderate stenosis group (1.975±0.074 ng/mL, 1.902±0.128 ng/mL, respectively, *P*<0.0001, Fig 3C). The difference of serum SM22α levels between patients with AAD and CAS indicates that SM22α is specifically released into peripheral blood in patients with AAD.

### Diagnostic value and correlation of serum SM22α levels with clinical parameters in patients with AAD and CAS

According to the ROC curve results, the AUC of serum SM22α for predicting AAD was 0.996, *P*<0.0001, sensitivity and specificity were 100% and 95%, respectively, the cut-off value was 2.514 ng/mL. When the levels of serum SM22α are greater than or equal to 2.514 ng/mL, the risk of AAD significantly increases (Fig 3E). These results suggest that the serum levels of SM22α have potential to be used as diagnostic biomarkers for AAD. We also found that the AUC value, sensitivity, and specificity of serum SM22α for predicting CAS was 0.977, 85% and 100%, respectively, the cut-off value was 2.028 ng/mL (Fig 3F), when the levels of serum SM22α are lower than or equal to 2.028 ng/mL, the risk of CAS significantly increases, suggesting that the detection of serum SM22α level may be used for rapid screening of the risk of CAS, with high specificity.

Correlation analysis showed that serum SM22α level in patients with AAD was particularly associated with FPG (*r* = 0.350, *P*<0.05), TC (*r* = 0.309, *P*<0.05), HDL-c (*r* = -0.372, *P*<0.05, Table 2), while further multiple linear regression analysis basically ruled out the interference of those related indicators on the serum SM22α level of AAD patients (*P*>0.05, Table 3).

**Table 2:** Correlation analysis of serum SM22α level with clinical indicators in patients with AAD, CAS.

Items	AAD		CAS	
	*r*	*P*	*r*	*P*
Stenosis rate (%)	**—**	**—**	-0.939	<0.001
Gender (men/women)	0.262	0.067	0.035	0.362
Age (years)	0.119	0.250	0.329	<0.001
Smoking [n (%)]	0.219	0.106	-0.035	0.360
Drinking [n (%)]	0.175	0.161	-0.031	0.374
SBP (mmHg)	0.177	0.159	-0.028	0.389
DBP (mmHg)	0.121	0.248	-0.265	0.003
FPG (mmol/L)	0.350	0.021	-0.011	0.456
TG (mmol/L)	0.219	0.106	-0.283	0.002
TC (mmol/L)	0.309	0.038	-0.021	0.416
HDL-c (mmol/L)	-0.372	0.015	0.290	0.001
LDL-c (mmol/L)	0.275	0.058	-0.150	0.061

*r*: Pearson’s correlation coefficient. Significance level: *P*<0.05.

**Table 3:** Multiple linear regression analysis of serum SM22α with clinical indicators in patients with AAD.

Indicators	Factors	*β*	SE	*β΄*	t	*P*
SM22α	FPG	0.090	0.069	0.229	1.307	0.201
	TC	0.045	0.057	0.141	0.794	0.434
	HDL-c	-0.156	0.098	-0.272	-1.594	0.121

Multivariate linear regression analysis with SM22α as dependent variable. Independent variables (FPG, TC and HDL-c) were obtained from correlation analysis. *β*: Partial regression coefficient, SE: Standard error of regression co- efficient, *β΄*: Standardized partial regression coefficient. Significance level: *P*<0.05.

In addition, as shown in Fig 3D and Table 2, serum SM22α levels in CAS patients were negatively correlated with the degree of carotid stenosis (*r* = -0.939, *P*<0.001), and there were also close correlations between serum SM22α and age (*r* = 0.329, *P*<0.001), DBP (*r* = -0.265, *P* = 0.003), TG (*r* = -0.283, *P* = 0.002), HDL-c (*r* = 0.290, *P* = 0.001) in patients with CAS. However, multiple linear regression analysis showed that only the degree of carotid artery stenosis was an independent related factor for serum SM22α level in patients with CAS (*P*<0.001, Table 4).

**Table 4:** Multiple linear regression analysis of serum SM22α with clinical indicators in patients with CAS.

Indicators	Factors	*β*	SE	*β΄*	t	*P*
SM22α	Stenosis rate	-0.010	<0.001	-0.948	-22.927	<0.001
	Age	<0.001	0.262	<0.001	-0.012	0.990
	DBP	<0.001	0.119	0.250	-0.446	0.642
	TG	0.360	0.219	0.106	0.306	0.760
	HDL-c	0.374	0.175	0.161	-0.806	0.422

Multivariate linear regression analysis with SM22α as dependent variable. Independent variables (stenosis rate, age, DBP, TG and HDL-c) were obtained from correlation analysis. *β*: Partial regression coefficient, SE: Standard error of regression coefficient, *β΄*: Standardized partial regression coefficient. Significance level: *P*<0.05.

## Discussion

Our studies and others have demonstrated that the disruption of SM22α impairs vascular structure and functions via promoting VSMC phenotype switching [7,14,19], whereas elevated SM22α expression also serves as a marker of VSMC senescence and hypertension [20]. Schellekens et al. found that plasma SM22α levels were closely paralleled the increasing degree of intestinal transmural damage upon progression of the duration of ischemia and a major part of the SM22α protein leaked out of the smooth muscle cells into blood [21]. However, this was not systemically studied by comparing serum, tissue and clinical data. So far, there is currently no specific serum biomarker of the media damage that might indicate VSMC phenotypic states and reflect the apoptosis of smooth muscle cells.

In animals, we demonstrate, for the first time, that SM22α serum levels are paralleled with its lower tissue expressions and may affect VSMC-specific contribution during the repair process after vascular injury, which drive damage of arterial media and reflect the progression of arterial media remodeling diseases. Importantly, the increased serum level of SM22α is associated with the occurrence of AAD. On the other hand, we confirmed that serum SM22α level in mice is negatively correlated with the rate of smooth muscle cells apoptosis in arterial media, the ratio of intima/media (I/M) area, atherosclerotic plaque size and extent. These data demonstrate that serum SM22α level as a specific biomarker in smooth muscle cells contributes to assess the structure and function of vascular media.

Typically, serum biomarkers should be potentially used to screen patients with compatible symptoms. Smooth muscle myosin heavy chain (SM-MHC) is a major component of medial smooth muscle, which is also present in uterine and intestinal smooth muscle [22]. Previous studies showed that in patients with aortic dissection admitted to the hospital within 24 hours of onset, SM-MHC levels in serum were greatly raised. At 24 hours after operation, however, all cases showed a rapid decrease to normal values [23,24]. Similarly, we reviewed 41 patients with AAD to examine the value of SM22α in diagnosing AAD. The results suggested that the serum level of SM22α was significantly increased in patients with AAD at admission and showed potential diagnostic value for AAD. That is, if patients with unexplained chest pain have high serum SM22α levels at admission, they may be at high risk of AAD and should receive aggressive monitoring and therapeutic interventions. In addition, our result obtained from the comparison of SM22α levels between AAD and CAS patients seems to be of particular interest, we found that serum SM22α levels were significantly increased in AAD patients compared with CAS patients and health controls, suggesting that SM22α is specifically released in patients at high risk of AAD. We also found that serum SM22α levels were markedly decreased at day 1 after surgery, and it was significantly reduced to the normal range on the 14th day after operation, suggesting that dynamic changes of serum SM22α level are potentially useful for a better understanding of AAD disease course and progression, meanwhile, they provide serological basis for the early intervention.

The following serum markers, in contrast, have its limitations. Elastin is one of the main structural components of the arterial wall. As one of the main pathological features of the aortic media in aortic dissection is elastin lamellar disruption, elastin degradation products (sELAF) could potentially be released into the circulation at the time of aortic dissection, which may reflect the damage of vascular media [25]. Shinohara et al. indicated that high sELAF serum levels were directly associated with aortic dissection. But sELAF remain elevated for a period of 72 hours in patients with aortic dissection, therefore, it is not suitable for early screening [26].

In addition, during the past decade, several potential diagnosis-predictive markers, such as high sensitivity C-reactive protein (hs-CRP) [27–29], D- dimer (DD) [30–33], serum amyloid A (SAA) [34], relaxin 2 (RL2) [35], have been reported. However, those markers were lack of specificity though they have some features for the clinical preliminary assessment and the markers themself have some factors suffering disturbance.

Serum-based SM22α tests are potentially more acceptable for screening of the general and high-risk population, as they are high specificity and sensitivity. Although our biomarker model based on the serum SM22α levels showed high performance in distinguishing arterial injury patients from the controls, there were still some limitations to our study. First, baseline levels of serum SM22α in normal controls, and the elevation of serum SM22α in AAD patients should be further validated with a large number of patients. Second, the causality of SM22α and other additional markers, especially those relating to inflammation and endothelial function, could not be inferred, the simultaneous measurement of other additional markers might expand our understanding. Third, we did not detect dynamic changes in plasma SM22α levels in the AAD patients. Finally, association between serum SM22α levels with in-hospital death needs to be confirmed in a large-scale study, as we had no death events in 41 AAD patients. We did not follow-up patients to assess long-term mortality or prognosis, either. Thus, we need to conduct follow-up studies to detect the clinical outcomes of these patients.

In conclusion, this study shows that serum SM22α level could serve as a potential biomarker for discerning AAD patients from CAS patients and the healthy population.

## Supporting information

S1 Table Changes of serum SM22α levels before and after operation. (DOCX)

S1 Dataset Dataset of the study. (DOCX)

## References

[1] Nienaber CA, Clough RE, Sakalihasan N, Suzuki T, Gibbs R, Mussa F, et al. Aortic dissection. Nat Rev Dis Primers. 2016; 2: 16053. doi: 10.1038/nrdp.2016.53 27440162

[2] Ranasinghe AM, Bonser RS. Biomarkers in acute aortic dissection and other aortic syndromes. J Am Coll Cardiol. 2010; 56: 1535–1541. doi: 10.1016/j.jacc.2010.01.076 21029872

[3] Goldfinger JZ, Halperin JL, Marin ML, Stewart AS, Eagle KA, Fuster V. Thoracic aortic aneurysm and dissection. J Am Coll Cardiol. 2014; 64: 1725–1739. doi: 10.1016/j.jacc.2014.08.025 25323262

[4] Luo F, Zhou XL, Li JJ, Hui RT. Inflammatory response is associated with aortic dissection. Ageing Res Rev. 2009; 8: 31–35. doi: 10.1016/j.arr.2008.08.001 18789403

[5] Michel JB, Jondeau G, Milewicz DM. From genetics to response to injury: vascular smooth muscle cells in aneurysms and dissections of the ascending aorta. Cardiovasc Res. 2018; 114: 578–589. doi: 10.1093/cvr/cvy006 29360940 PMC6658716

[6] Owens GK, Kumar MS, Wamhoff BR. Molecular regulation of vascular smooth muscle cell differentiation in development and disease. Physiol Rev. 2004; 84: 767–801. doi: 10.1152/physrev.00041.2003 15269336

[7] López-Candales A, Holmes DR, Liao S, Scott MJ, Wickline SA, Thompson RW. Decreased vascular smooth muscle cell density in medial degeneration of human abdominal aortic aneurysms. Am J Pathol. 1997; 150: 993–1007. 9060837 PMC1857880

[8] Wamhoff BR, Hoofnagle MH, Burns A, Sinha S, McDonald OG, Owens GK. A G/C element mediates repression of the SM22 alpha promoter within phenotypically modulated smooth muscle cells in experimental athero-sclerosis. Circ Res, 2004; 95: 981–988. doi: 10.1161/01.RES.0000147961.09840.fb 15486317

[9] Feil S, Fehrenbacher B, Lukowski R, Essmann F, Schulze-Osthoff K, Schaller M, et al. Transdifferentiation of vascular smooth muscle cells to macrophage-like cells during atherogenesis. Circ Res. 2014; 115: 662–667. doi: 10.1161/CIRCRESAHA.115.304634 25070003

[10] Zhong L, He X, Si X, Wang H, Li B, Hu Y, et al. SM22α (Smooth Muscle 22α) prevents aortic aneurysm formation by inhibiting smooth muscle cell phenotypic switching through suppressing reactive oxygen species/NF-κB. Arterioscler Thromb Vasc Biol. 2019; 39: e10–e25. doi: 10.1161/ATVBAHA.118.311917 30580562

[11] Ailawadi G, Moehle CW, Pei H, Walton SP, Yang Z, Kron IL, et al. Smooth muscle phenotypic modulation is an early event in aortic aneurysms. J Thorac Cardiovasc Surg. 2009; 138: 1392–1399. doi: 10.1016/j.jtcvs.2009.07.075 19931668 PMC2956879

[12] Zhao Z, Wang Y, Li S, Liu S, Liu Y, Yu Y, et al. HSP90 inhibitor 17-DMAG effectively alleviated the progress of thoracic aortic dissection by suppressing smooth muscle cell phenotypic switch. Am J Transl Res. 2019; 11: 509–518. 30788006 PMC6357309

[13] Shen J, Yang M, Ju D, Jiang H, Zheng JP, Xu Z, et al. Disruption of SM22 promotes inflammation after artery injury via nuclear factor kappaB activation. Circ Res. 2010; 106: 1351–1362. doi: 10.1161/CIRCRESAHA.109.213900 20224039 PMC2896867

[14] Zhao G, Fu Y, Cai Z, Yu F, Gong Z, Dai R, et al. Unspliced XBP1 confers VSMC homeostasis and prevents aortic aneurysm formation via FoxO4 interaction. Circ Res. 2017; 121: 1331–1345. doi: 10.1161/CIRCRESAHA.117.311450 29089350

[15] Lv P, Miao SB, Shu YN, Dong LH, Liu G, Xie XL, et al. Phosphorylation of smooth muscle 22α facilitates angiotensin II-induced ROS production via activation of the PKCδ-P47^phox^ axis through release of PKCδ and actin dynamics and is associated with hypertrophy and hyperplasia of vascular smooth muscle cells in vitro and in vivo. Circ Res. 2012; 111: 697–707. doi: 10.1161/CIRCRESAHA.112.272013 22798525

[16] Shu YN, Zhang F, Bi W, Dong LH, Zhang DD, Chen R, et al. SM22α inhibits vascular inflammation via stabilization of IκBα in vascular smooth muscle cells. J Mol Cell Cardiol. 2015; 84: 191–199. doi: 10.1016/j.yjmcc.2015.04.020 25937534

[17] Shu YN, Dong LH, Li H, Pei QQ, Miao SB, Zhang F, et al. CKII-SIRT1-SM22α loop evokes a self-limited inflammatory response in vascular smooth muscle cells. Cardiovasc Res. 2017; 113: 1198–1207. doi: 10.1093/cvr/cvx048 28419207

[18] Lv P, Yin YJ, Kong P, Cao L, Xi H, Wang N, et al. SM22α loss contributes to apoptosis of vascular smooth muscle cells via macrophage-derived circRasGEF1B. Oxid Med Cell Longev. 2021; 2021: 5564884. doi: 10.1155/2021/5564884 33859778 PMC8026322

[19] Kaplan-Albuquerque N, Garat C, Van Putten V, Nemenoff RA. Regulation of SM22α expression by arginine vasopressin and PDGF-BB in vascular smooth muscle cells. Am J Physiol Heart Circ Physiol. 2003; 285: H1444–H1452. doi: 10.1152/ajpheart.00306.2003 12829429

[20] Miao SB, Xie XL, Yin YJ, Zhao LL, Zhang F, Shu YN, et al. Accumulation of smooth muscle 22α protein accelerates senescence of vascular smooth muscle cells via stabilization of p53 in vitro and in vivo. Arterioscler Thromb Vasc Biol. 2017; 37: 1849–1859. doi: 10.1161/ATVBAHA.117.309378 28798142

[21] Schellekens DHSM, Reisinger KW, Lenaerts K, Hadfoune M, Olde Damink SW, Buurman WA, et al. SM22α Plasma Biomarker for Human Transmural Intestinal Ischemia. Ann Surg. 2018; 268: 120–126. doi: 10.1097/SLA.0000000000002278 28525410

[22] Suzuki T, Katoh H, Watanabe M, Kurabayashi M, Hiramori K, Hori S, et al. Novel biochemical diagnostic method for aortic dissection. Results of a prospective study using an immunoassay of smooth muscle myosin heavy chain. Circulation. 1996; 93: 1244–1249. doi: 10.1161/01.cir.93.6.1244 8653847

[23] Katoh H, Suzuki T, Hiroi Y, Ohtaki E, Suzuki S, Yazaki Y, et al. Diagnosis of aortic dissection by immunoassay for circulating smooth muscle myosin. Lancet. 1995; 345: 191–192. doi: 10.1016/s0140-6736(95)90194-9 7823685

[24] Katoh H, Suzuki T, Yokomori K, Suzuki S, Ohtaki E, Watanabe M, et al. A novel immunoassay of smooth muscle myosin heavy chain in serum. J Immunol Methods. 1995; 185: 57–63. doi: 10.1016/0022-1759(95)00104-i 7665900

[25] Olin JW, Fuster V. Acute aortic dissection: the need for rapid, accurate, and readily available diagnostic strategies. Arterioscler Thromb Vasc Biol. 2003; 23: 1721–1723. doi: 10.1161/01.ATV.0000093222.33222.D2 14555642

[26] Shinohara T, Suzuki K, Okada M, Shigai M, Shimizu M, Maehara T, et al. Soluble elastin fragments in serum are elevated in aortic dissection. J Cardiol. 2004; 43: 96–97. 15046049

[27] Huang X, Wang A, Liu X, Chen S, Zhu Y, Liu Y, et al. Association between high sensitivity C-reactive protein and prevalence of asymptomatic carotid artery stenosis. Atherosclerosis. 2016; 246: 44–49. doi: 10.1016/j.atherosclerosis.2015.12.024 26752692

[28] Schillger M, Domanovits H, Bayegan K, Hölzenbein T, Grabenwöger M, Thoenissen J, et al. C-reactive protein and mortality in patients with acute aortic disease. Intensive Care Med. 2002; 28: 740–745. doi: 10.1007/s00134-002-1299-1 12107680

[29] Shangwei Z, Yingqi W, Jiang X, Zhongyin W, Juan J, Dafang C, et al. Serum high-sensitive C-reactive protein level and CRP genetic poly-morphisms are associated with abdominal aortic aneurysm. Ann Vasc Surg. 2017; 45: 186–192. doi: 10.1016/j.avsg.2017.05.024 28549956

[30] Eggebrecht H, Naber CK, Bruch C, Kröger K, von Birgelen C, Schmermund A, et al. Value of plasma fibrin D-dimers for detection of acute aortic dissection. J Am Coll Cardiol. 2004; 44: 804–809. doi: 10.1016/j.jacc.2004.04.053 15312863

[31] Weber T, Högler S, Auer J, Berent R, Lassnig E, Kvas E, et al. D-dimer in acute aortic dissection. Chest. 2003; 123: 1375–1378. doi: 10.1378/chest.123.5.1375 12740250

[32] Vele E, Kurtcehajic A, Zerem E, Maskovic J, Alibegovic E, Hujdurovic A. Plasma D-dimer as a predictor of the progression of abdominal aortic aneurysm. J Thromb Haemost. 2016; 14: 2298–2303. doi: 10.1111/jth.13487 27567003

[33] Fan YN, Ke X, Yi ZL, Lin YQ, Deng BQ, Shu XR, et al. Plasma D-dimer as a predictor of intraluminal thrombus burden and progression of abdominal aortic aneurysm. Life Sci. 2020; 240: 117069. doi: 10.1016/j.lfs.2019.117069 31751582

[34] He Y, Ma C, Xing J, Wang S, Ji C, Han Y, et al. Serum amyloid a protein as a potential biomarker in predicting acute onset and association with in-hospital death in acute aortic dissection. BMC Cardiovasc Disord. 2019; 19: 282. doi: 10.1186/s12872-019-1267-0 31810459 PMC6898938

[35] Papoutsis K, Kapelouzou A, Tsilimigras DI, Patelis N, Kouvelos G, Schizas D, et al. Associations between serum relaxin 2, aneurysm formation/size and severity of atherosclerosis: a preliminary prospective analysis. Acta Pharmacol Sin. 2018; 39: 1243–1248. doi: 10.1038/aps.2018.8 29565035 PMC6289330

