# Supplementary material for: Assessing serum levels of SM22α as a new biomarker for patients with aortic aneurysm/dissection
Source: PLoS One. 2022 Mar 31;17(3):e0264942. doi: 10.1371/journal.pone.0264942 (PMC8970406; doi:10.1371/journal.pone.0264942)
Supplement: S1 Table — (DOCX) [file pone.0264942.s001.docx]

**Table S2 Changes of serum SM22α levels before and after operation**

| **Patients ID** | **Time of postoperative recovery (days)** | | | | |
| --- | --- | --- | --- | --- | --- |
|  | **0** | **1** | **5** | **7** | **14** |
| No.41 | 2.96 | 2.79 | 2.63 | 2.47 | 2.20 |
| No.42 | 2.60 | 2.41 | 2.30 | 2.21 | 2.21 |
| No.43 | 3.11 | 2.76 | 2.01 | 2.22 | 2.11 |
| No.44 | 3.60 | 2.43 | 2.19 | 2.82 | 2.07 |
| No.45 | 3.07 | 2.54 | 2.26 | 2.56 | 2.16 |
| No.46 | 2.96 | 3.03 | 2.59 | 2.59 | 2.32 |
| No.47 | 3.39 | 2.34 | 1.96 | 2.33 | 2.06 |
| No.48 | 3.56 | 3.42 | 2.07 | 2.25 | 2.05 |
| No.49 | 3.32 | 2.52 | 2.49 | 2.11 | 2.12 |
| No.50 | 3.16 | 2.26 | 1.92 | 2.04 | 2.05 |
| No.51 | 3.39 | 2.77 | 2.64 | 1.99 | 2.04 |
| No.52 | 3.65 | 2.54 | 2.61 | 1.88 | 2.24 |
| No.53 | 3.32 | 2.08 | 2.84 | 2.56 | 2.01 |

Serum samples from 13 patients with type A dissection were collected.
