# Supplementary material for: Assessing serum levels of SM22α as a new biomarker for patients with aortic aneurysm/dissection
Source: PLoS One. 2022 Mar 31;17(3):e0264942. doi: 10.1371/journal.pone.0264942 (PMC8970406; doi:10.1371/journal.pone.0264942)
Supplement: S1 Dataset — (DOCX) [file pone.0264942.s002.docx]

**Table S1 Dataset of the study**

| **ID** | **Serum SM22α** | **Sex** | **Age** | **Smoking** | **Drinking** | **SBP** | **DBP** | **FPG** | **TG** | **TC** | **HDL** | **LDL** | **Stenosis rate (%)** |
| --- | --- | --- | --- | --- | --- | --- | --- | --- | --- | --- | --- | --- | --- |
| No.1 | 2.16 | M | 68 | No | No | 139 | 70 | 5.28 | 1.83 | 5.28 | 1.34 | 3.55 | — |
| No.2 | 2.28 | M | 64 | No | No | 145 | 80 | 5.67 | 1.48 | 4.81 | 1.23 | 3.33 | — |
| No.3 | 2.46 | F | 64 | No | No | 140 | 80 | 9.20 | 0.84 | 4.88 | 1.50 | 3.28 | — |
| No.4 | 2.19 | M | 64 | No | No | 120 | 60 | 4.70 | 1.82 | 4.57 | 1.23 | 3.07 | — |
| No.5 | 2.10 | M | 63 | No | No | 140 | 90 | 4.85 | 1.46 | 3.85 | 1.09 | 2.65 | — |
| No.6 | 2.26 | F | 63 | No | No | 145 | 90 | 4.52 | 1.39 | 5.68 | 1.59 | 3.85 | — |
| No.7 | 2.37 | M | 63 | No | No | 135 | 76 | 4.60 | 2.52 | 3.98 | 0.88 | 2.60 | — |
| No.8 | 2.19 | M | 63 | No | No | 146 | 80 | 5.13 | 2.55 | 5.02 | 1.23 | 3.34 | — |
| No.9 | 2.20 | M | 62 | No | No | 135 | 77 | 4.32 | 1.52 | 4.94 | 1.22 | 3.15 | — |
| No.10 | 2.13 | F | 62 | No | No | 142 | 80 | 5.13 | 2.90 | 3.78 | 1.11 | 2.22 | — |
| No.11 | 2.68 | F | 62 | No | No | 136 | 76 | 4.63 | 1.19 | 5.95 | 1.62 | 4.29 | — |
| No.12 | 2.34 | M | 62 | Yes | Yes | 140 | 70 | 4.34 | 1.62 | 4.65 | 1.10 | 3.12 | — |
| No.13 | 2.28 | F | 62 | No | No | 138 | 66 | 4.87 | 0.94 | 4.65 | 2.44 | 1.78 | — |
| No.14 | 2.18 | F | 61 | No | No | 138 | 60 | 4.94 | 0.88 | 6.53 | 2.22 | 4.08 | — |
| No.15 | 2.03 | M | 60 | No | No | 144 | 80 | 6.03 | 1.24 | 5.61 | 1.88 | 3.41 | — |
| No.16 | 2.06 | M | 58 | No | No | 140 | 70 | 4.88 | 1.79 | 5.63 | 1.45 | 3.60 | — |
| No.17 | 2.07 | M | 58 | Yes | Yes | 138 | 65 | 6.50 | 2.50 | 5.04 | 1.17 | 3.12 | — |
| No.18 | 2.51 | M | 58 | No | No | 144 | 88 | 5.61 | 3.79 | 4.59 | 0.88 | 2.57 | — |
| No.19 | 2.31 | F | 58 | No | No | 138 | 76 | 7.26 | 1.73 | 5.59 | 1.62 | 3.40 | — |
| No.20 | 2.44 | M | 57 | No | No | 130 | 60 | 6.52 | 0.62 | 3.94 | 1.29 | 2.49 | — |
| No.21 | 2.04 | F | 57 | No | No | 135 | 65 | 5.01 | 3.60 | 6.33 | 1.20 | 4.07 | — |
| No.22 | 2.22 | M | 57 | Yes | No | 140 | 80 | 4.87 | 1.61 | 4.29 | 1.08 | 2.97 | — |
| No.23 | 2.21 | F | 56 | No | No | 150 | 90 | 4.79 | 1.02 | 5.35 | 1.45 | 3.22 | — |
| No.24 | 2.04 | F | 56 | No | No | 148 | 88 | 4.64 | 1.47 | 5.24 | 1.20 | 3.64 | — |
| No.25 | 2.07 | F | 56 | No | No | 136 | 70 | 4.79 | 0.78 | 4.86 | 1.75 | 3.04 | — |
| No.26 | 2.47 | F | 49 | No | No | 150 | 80 | 4.86 | 0.87 | 5.91 | 1.62 | 3.93 | — |
| No.27 | 2.38 | F | 49 | No | No | 144 | 70 | 4.46 | 0.89 | 4.76 | 1.43 | 3.08 | — |
| No.28 | 2.11 | M | 46 | No | No | 136 | 70 | 5.00 | 1.05 | 3.89 | 1.08 | 2.68 | — |
| No.29 | 2.10 | F | 46 | No | No | 120 | 60 | 5.54 | 1.70 | 4.41 | 1.28 | 2.80 | — |
| No.30 | 2.21 | F | 44 | No | No | 130 | 70 | 4.69 | 0.84 | 5.45 | 1.53 | 3.55 | — |
| No.31 | 2.36 | M | 43 | Yes | No | 134 | 73 | 4.62 | 1.29 | 6.61 | 1.30 | 4.94 | — |
| No.32 | 2.06 | M | 42 | No | Yes | 150 | 90 | 4.66 | 0.64 | 4.53 | 1.39 | 3.06 | — |
| No.33 | 2.20 | F | 42 | No | No | 140 | 80 | 4.49 | 0.73 | 3.60 | 1.20 | 2.20 | — |
| No.34 | 2.14 | F | 40 | No | No | 135 | 75 | 4.23 | 1.35 | 5.04 | 2.25 | 2.31 | — |
| No.35 | 2.08 | M | 39 | No | Yes | 130 | 65 | 4.46 | 0.87 | 4.04 | 1.40 | 2.44 | — |
| No.36 | 2.53 | M | 39 | No | No | 120 | 60 | 4.94 | 1.10 | 4.67 | 1.23 | 3.33 | — |
| No.37 | 2.33 | M | 38 | No | No | 130 | 65 | 4.51 | 3.84 | 4.96 | 1.02 | 2.99 | — |
| No.38 | 2.48 | M | 35 | Yes | Yes | 133 | 70 | 4.73 | 1.81 | 4.76 | 1.18 | 3.25 | — |
| No.39 | 2.21 | F | 35 | No | No | 140 | 90 | 4.48 | 0.63 | 3.85 | 1.39 | 2.36 | — |
| No.40 | 2.18 | F | 36 | No | No | 134 | 80 | 5.28 | 0.75 | 3.41 | 0.97 | 2.36 | — |
| No.41 | 2.96 | M | 61 | No | No | 157 | 90 | 5.78 | 3.12 | 4.14 | 1.82 | 2.01 | — |
| No.42 | 2.60 | F | 60 | No | No | 148 | 70 | 5.55 | 3.11 | 5.59 | 2.05 | 1.88 | — |
| No.43 | 3.11 | M | 38 | No | No | 160 | 98 | 3.12 | 3.18 | 4.36 | 0.58 | 1.31 | — |
| No.44 | 3.60 | M | 52 | Yes | Yes | 154 | 96 | 5.23 | 2.88 | 4.45 | 1.17 | 3.96 | — |
| No.45 | 3.07 | M | 66 | Yes | Yes | 153 | 87 | 5.51 | 3.72 | 3.54 | 0.97 | 1.99 | — |
| No.46 | 2.96 | M | 43 | No | No | 150 | 90 | 3.21 | 3.00 | 1.87 | 2.49 | 3.95 | — |
| No.47 | 3.39 | M | 67 | No | No | 163 | 90 | 4.43 | 3.21 | 4.34 | 1.44 | 4.00 | — |
| No.48 | 3.56 | M | 66 | No | No | 141 | 89 | 5.36 | 3.57 | 3.93 | 0.74 | 1.59 | — |
| No.49 | 3.32 | M | 42 | No | No | 146 | 87 | 5.56 | 2.83 | 2.46 | 2.30 | 3.79 | — |
| No.50 | 3.16 | F | 78 | No | Yes | 159 | 80 | 4.59 | 3.75 | 3.24 | 0.92 | 4.18 | — |
| No.51 | 3.39 | M | 47 | Yes | Yes | 158 | 90 | 4.23 | 3.26 | 2.95 | 1.79 | 3.25 | — |
| No.52 | 3.65 | M | 40 | No | No | 130 | 78 | 5.12 | 3.09 | 3.93 | 2.10 | 4.30 | — |
| No.53 | 3.32 | F | 65 | No | No | 158 | 85 | 4.67 | 3.24 | 2.45 | 1.22 | 3.45 | — |
| No.54 | 3.34 | M | 38 | Yes | Yes | 149 | 63 | 5.31 | 3.20 | 3.98 | 0.61 | 2.31 | — |
| No.55 | 2.98 | M | 63 | No | No | 147 | 70 | 5.11 | 3.20 | 2.37 | 1.80 | 1.80 | — |
| No.56 | 2.52 | F | 55 | No | No | 140 | 90 | 3.09 | 2.89 | 2.08 | 2.24 | 1.65 | — |
| No.57 | 2.52 | M | 33 | No | No | 145 | 75 | 5.23 | 3.28 | 2.64 | 1.48 | 3.84 | — |
| No.58 | 3.33 | M | 65 | Yes | Yes | 145 | 70 | 4.45 | 3.88 | 3.10 | 0.62 | 2.46 | — |
| No.59 | 3.44 | M | 72 | No | Yes | 150 | 78 | 4.78 | 3.97 | 4.60 | 1.13 | 2.50 | — |
| No.60 | 3.30 | M | 65 | No | No | 165 | 90 | 5.02 | 3.04 | 2.06 | 0.76 | 3.95 | — |
| No.61 | 2.71 | F | 54 | No | No | 155 | 86 | 3.51 | 2.04 | 1.82 | 1.54 | 1.45 | — |
| No.62 | 3.19 | M | 55 | Yes | Yes | 167 | 90 | 4.55 | 1.73 | 4.60 | 0.93 | 4.43 | — |
| No.63 | 3.62 | M | 45 | No | No | 157 | 85 | 6.01 | 3.30 | 4.59 | 0.92 | 2.65 | — |
| No.64 | 2.65 | M | 47 | No | No | 160 | 90 | 4.12 | 3.10 | 1.95 | 2.04 | 1.16 | — |
| No.65 | 3.44 | M | 36 | No | No | 167 | 75 | 4.33 | 4.90 | 2.56 | 2.07 | 2.48 | — |
| No.66 | 3.54 | F | 69 | No | No | 178 | 90 | 5.67 | 3.66 | 3.40 | 1.19 | 2.75 | — |
| No.67 | 3.17 | M | 58 | Yes | No | 155 | 70 | 5.98 | 3.19 | 2.71 | 0.66 | 4.05 | — |
| No.68 | 3.33 | M | 58 | No | No | 168 | 90 | 5.32 | 3.06 | 2.67 | 0.89 | 2.73 | — |
| No.69 | 3.12 | M | 34 | No | No | 153 | 67 | 3.34 | 4.94 | 2.91 | 1.33 | 3.78 | — |
| No.70 | 3.11 | M | 35 | No | No | 154 | 80 | 5.12 | 3.86 | 2.41 | 1.40 | 2.13 | — |
| No.71 | 3.04 | F | 57 | No | No | 143 | 67 | 4.87 | 3.48 | 3.93 | 0.70 | 2.22 | — |
| No.72 | 3.44 | F | 55 | No | No | 150 | 90 | 4.59 | 3.03 | 2.35 | 1.19 | 1.53 | — |
| No.73 | 3.58 | M | 62 | No | No | 155 | 80 | 5.09 | 3.15 | 3.90 | 0.93 | 1.65 | — |
| No.74 | 2.99 | M | 65 | No | No | 150 | 80 | 4.17 | 2.89 | 2.26 | 1.25 | 1.53 | — |
| No.75 | 2.71 | M | 68 | Yes | Yes | 150 | 80 | 4.51 | 3.82 | 4.66 | 1.58 | 2.65 | — |
| No.76 | 2.40 | M | 75 | No | No | 144 | 79 | 4.66 | 1.84 | 3.39 | 2.62 | 1.67 | — |
| No.77 | 2.47 | F | 68 | No | No | 160 | 90 | 3.12 | 2.14 | 2.38 | 2.49 | 1.11 | — |
| No.78 | 2.60 | M | 48 | Yes | No | 143 | 78 | 3.98 | 2.05 | 3.62 | 1.09 | 1.96 | — |
| No.79 | 2.70 | M | 61 | Yes | Yes | 160 | 90 | 3.91 | 2.38 | 3.03 | 1.40 | 2.88 | — |
| No.80 | 2.59 | M | 67 | No | No | 149 | 79 | 4.02 | 2.24 | 2.49 | 1.07 | 2.34 | — |
| No.81 | 2.70 | M | 39 | Yes | Yes | 150 | 90 | 5.41 | 3.56 | 3.84 | 2.53 | 1.48 | — |
| No.82 | 1.45 | F | 66 | No | No | 149 | 80 | 2.70 | 4.45 | 2.49 | 1.15 | 1.83 | 95 |
| No.83 | 1.50 | M | 52 | No | Yes | 135 | 75 | 3.26 | 1.38 | 3.11 | 0.71 | 4.01 | 95 |
| No.84 | 1.46 | M | 72 | No | No | 141 | 72 | 3.51 | 4.78 | 2.41 | 0.79 | 3.35 | 95 |
| No.85 | 1.40 | M | 52 | Yes | Yes | 146 | 99 | 4.47 | 1.16 | 3.15 | 1.03 | 1.62 | 95 |
| No.86 | 1.45 | M | 47 | No | No | 138 | 85 | 4.34 | 2.74 | 5.85 | 1.02 | 4.48 | 95 |
| No.87 | 1.46 | F | 56 | No | No | 140 | 75 | 4.49 | 2.74 | 3.31 | 1.46 | 4.55 | 95 |
| No.88 | 1.53 | F | 55 | No | No | 146 | 81 | 4.38 | 4.23 | 2.27 | 1.22 | 5.32 | 92 |
| No.89 | 1.46 | M | 68 | No | No | 133 | 75 | 4.74 | 2.16 | 3.27 | 1.08 | 3.19 | 92 |
| No.90 | 1.61 | M | 55 | No | No | 156 | 109 | 5.73 | 0.80 | 2.05 | 0.97 | 0.72 | 92 |
| No.91 | 1.54 | M | 49 | No | No | 121 | 69 | 5.34 | 2.40 | 1.47 | 1.17 | 0.89 | 92 |
| No.92 | 1.50 | F | 60 | No | No | 144 | 78 | 4.23 | 4.34 | 4.18 | 1.44 | 4.20 | 90 |
| No.93 | 1.63 | M | 71 | Yes | No | 142 | 78 | 4.60 | 4.56 | 3.91 | 1.27 | 3.34 | 90 |
| No.94 | 1.55 | M | 47 | No | No | 175 | 101 | 13.57 | 4.75 | 4.01 | 1.27 | 1.05 | 90 |
| No.95 | 1.43 | M | 62 | Yes | Yes | 130 | 71 | 5.88 | 1.44 | 3.69 | 1.21 | 2.13 | 90 |
| No.96 | 1.58 | M | 63 | No | No | 107 | 81 | 4.33 | 5.86 | 4.21 | 1.10 | 3.35 | 90 |
| No.97 | 1.54 | F | 73 | No | No | 137 | 76 | 5.66 | 1.67 | 4.58 | 1.26 | 2.69 | 90 |
| No.98 | 1.44 | M | 43 | Yes | No | 104 | 61 | 4.74 | 5.88 | 6.88 | 1.15 | 1.35 | 90 |
| No.99 | 1.60 | M | 56 | Yes | No | 168 | 80 | 6.86 | 5.57 | 5.28 | 0.95 | 3.14 | 89 |
| No.100 | 1.45 | M | 62 | No | No | 133 | 77 | 4.71 | 3.98 | 5.87 | 1.12 | 2.01 | 89 |
| No.101 | 1.61 | F | 66 | No | No | 177 | 87 | 8.37 | 3.27 | 4.41 | 0.96 | 2.12 | 89 |
| No.102 | 1.50 | M | 69 | No | No | 142 | 85 | 5.04 | 4.39 | 4.88 | 1.13 | 3.36 | 89 |
| No.103 | 1.54 | F | 54 | No | No | 141 | 86 | 4.80 | 3.01 | 5.71 | 1.01 | 1.38 | 89 |
| No.104 | 1.56 | M | 60 | No | No | 105 | 76 | 5.09 | 4.79 | 6.68 | 0.78 | 0.64 | 85 |
| No.105 | 1.64 | F | 65 | No | No | 145 | 75 | 6.88 | 1.39 | 6.01 | 1.13 | 1.55 | 85 |
| No.106 | 1.64 | F | 71 | No | No | 142 | 82 | 5.92 | 2.29 | 3.19 | 1.07 | 4.43 | 85 |
| No.107 | 1.58 | M | 54 | Yes | Yes | 114 | 94 | 6.11 | 0.99 | 3.52 | 1.21 | 0.83 | 85 |
| No.108 | 1.61 | M | 67 | No | No | 135 | 84 | 4.76 | 1.07 | 3.47 | 1.18 | 5.97 | 85 |
| No.109 | 1.63 | M | 54 | No | No | 141 | 99 | 4.44 | 1.37 | 5.31 | 1.23 | 3.15 | 85 |
| No.110 | 1.63 | M | 39 | Yes | No | 132 | 84 | 3.51 | 1.35 | 3.23 | 0.92 | 5.93 | 85 |
| No.111 | 1.63 | M | 77 | No | No | 118 | 74 | 4.51 | 2.22 | 3.53 | 1.21 | 2.35 | 85 |
| No.112 | 1.63 | M | 52 | No | No | 126 | 78 | 6.15 | 1.84 | 3.76 | 1.22 | 2.11 | 85 |
| No.113 | 1.67 | F | 70 | No | No | 114 | 72 | 16.3 | 2.15 | 4.29 | 1.49 | 2.44 | 85 |
| No.114 | 1.73 | M | 58 | No | No | 146 | 100 | 5.32 | 2.09 | 4.10 | 0.99 | 2.36 | 85 |
| No.115 | 1.67 | M | 53 | No | No | 125 | 84 | 4.27 | 1.48 | 3.18 | 0.97 | 5.70 | 85 |
| No.116 | 1.60 | M | 67 | No | No | 136 | 91 | 4.66 | 2.67 | 2.24 | 0.92 | 5.18 | 80 |
| No.117 | 1.62 | F | 58 | No | No | 135 | 105 | 11.12 | 1.98 | 6.46 | 1.07 | 4.91 | 80 |
| No.118 | 1.68 | M | 72 | No | No | 150 | 80 | 5.64 | 2.99 | 3.45 | 0.81 | 2.33 | 80 |
| No.119 | 1.69 | M | 56 | No | No | 121 | 75 | 5.26 | 2.60 | 3.32 | 0.82 | 5.76 | 80 |
| No.120 | 1.71 | M | 69 | Yes | No | 126 | 84 | 3.81 | 1.61 | 3.53 | 1.01 | 2.13 | 75 |
| No.121 | 1.76 | M | 61 | No | No | 146 | 78 | 8.51 | 3.28 | 3.53 | 0.83 | 2.19 | 72 |
| No.122 | 2.01 | F | 78 | No | No | 182 | 90 | 5.59 | 2.92 | 6.91 | 5.18 | 1.42 | 50 |
| No.123 | 2.17 | M | 90 | No | No | 139 | 61 | 6.34 | 2.62 | 6.38 | 1.21 | 1.36 | 50 |
| No.124 | 2.10 | F | 79 | No | No | 134 | 74 | 5.19 | 3.85 | 6.02 | 1.55 | 4.89 | 50 |
| No.125 | 1.95 | M | 70 | No | No | 115 | 65 | 5.24 | 2.31 | 4.41 | 1.01 | 2.85 | 50 |
| No.126 | 2.05 | M | 66 | No | No | 123 | 60 | 6.54 | 2.79 | 3.89 | 1.16 | 2.04 | 50 |
| No.127 | 2.03 | M | 64 | No | No | 133 | 65 | 5.35 | 3.10 | 4.22 | 1.14 | 4.65 | 50 |
| No.128 | 2.02 | M | 67 | No | No | 142 | 77 | 6.04 | 5.52 | 4.27 | 1.07 | 2.27 | 50 |
| No.129 | 2.10 | M | 55 | No | No | 125 | 80 | 18.00 | 2.43 | 5.63 | 1.53 | 1.08 | 50 |
| No.130 | 2.15 | M | 84 | No | No | 151 | 60 | 6.92 | 2.64 | 3.71 | 1.08 | 2.47 | 50 |
| No.131 | 2.08 | F | 75 | No | No | 123 | 58 | 5.64 | 1.95 | 3.95 | 1.12 | 2.12 | 51 |
| No.132 | 1.85 | M | 57 | Yes | Yes | 144 | 78 | 5.17 | 1.32 | 5.41 | 1.41 | 3.91 | 53 |
| No.133 | 1.84 | M | 83 | No | No | 123 | 70 | 4.91 | 1.50 | 3.01 | 0.87 | 4.53 | 54 |
| No.134 | 1.91 | M | 67 | No | No | 151 | 80 | 5.55 | 1.75 | 5.73 | 1.33 | 4.00 | 55 |
| No.135 | 1.82 | M | 70 | No | No | 140 | 90 | 4.80 | 0.91 | 3.30 | 1.17 | 4.78 | 55 |
| No.136 | 1.95 | F | 67 | No | No | 135 | 80 | 6.35 | 1.32 | 6.16 | 1.70 | 4.20 | 55 |
| No.137 | 1.84 | M | 85 | No | No | 142 | 89 | 5.08 | 1.91 | 4.30 | 1.03 | 2.65 | 59 |
| No.138 | 1.90 | M | 65 | No | No | 112 | 60 | 5.04 | 2.37 | 6.24 | 1.14 | 4.00 | 59 |
| No.139 | 1.94 | M | 74 | No | No | 120 | 60 | 3.63 | 1.59 | 5.31 | 1.42 | 3.59 | 60 |
| No.140 | 1.82 | M | 82 | No | No | 157 | 88 | 3.55 | 1.22 | 4.93 | 1.06 | 3.40 | 60 |
| No.141 | 1.73 | M | 85 | No | Yes | 159 | 77 | 4.39 | 2.94 | 4.50 | 1.27 | 4.82 | 62 |
| No.142 | 1.82 | M | 79 | No | Yes | 175 | 107 | 6.38 | 1.80 | 7.06 | 1.35 | 2.19 | 62 |
| No.143 | 1.86 | F | 83 | Yes | Yes | 155 | 72 | 4.85 | 1.43 | 4.38 | 1.48 | 2.45 | 64 |
| No.144 | 1.73 | M | 79 | No | No | 142 | 68 | 3.93 | 1.20 | 4.92 | 1.17 | 3.24 | 64 |
| No.145 | 1.73 | M | 88 | No | No | 163 | 90 | 4.43 | 1.34 | 3.85 | 1.03 | 5.28 | 64 |
| No.146 | 1.81 | M | 62 | No | Yes | 110 | 81 | 3.29 | 1.68 | 4.76 | 0.98 | 3.23 | 64 |
| No.147 | 1.75 | F | 81 | No | No | 165 | 90 | 4.31 | 1.40 | 8.27 | 2.41 | 5.38 | 65 |
| No.148 | 1.73 | M | 65 | No | Yes | 156 | 80 | 4.81 | 1.55 | 4.94 | 1.32 | 3.14 | 65 |
| No.149 | 1.65 | M | 83 | No | Yes | 140 | 78 | 7.94 | 7.12 | 7.29 | 0.87 | 4.11 | 68 |
| No.150 | 1.92 | F | 58 | No | No | 147 | 80 | 6.38 | 2.00 | 4.39 | 1.15 | 1.76 | 60 |
| No.151 | 1.78 | M | 53 | No | No | 114 | 60 | 4.11 | 1.36 | 5.66 | 1.55 | 3.66 | 65 |
| No.152 | 2.05 | M | 52 | Yes | Yes | 137 | 93 | 4.80 | 3.10 | 5.13 | 1.19 | 1.46 | 50 |
| No.153 | 1.80 | F | 84 | Yes | No | 186 | 81 | 7.39 | 1.39 | 5.69 | 1.39 | 3.99 | 55 |
| No.154 | 1.89 | M | 83 | Yes | No | 97 | 60 | 5.87 | 0.80 | 4.16 | 1.71 | 1.84 | 60 |
| No.155 | 1.84 | M | 82 | No | No | 142 | 71 | 5.46 | 0.76 | 4.66 | 1.56 | 2.93 | 55 |
| No.156 | 1.96 | M | 80 | No | No | 165 | 92 | 5.54 | 0.95 | 4.92 | 1.59 | 3.25 | 55 |
| No.157 | 2.00 | M | 61 | Yes | Yes | 140 | 77 | 5.27 | 1.16 | 5.17 | 1.09 | 3.98 | 50 |
| No.158 | 1.91 | M | 61 | No | Yes | 147 | 82 | 5.11 | 1.16 | 5.63 | 1.46 | 2.07 | 54 |
| No.159 | 1.83 | F | 67 | Yes | No | 135 | 65 | 4.92 | 3.83 | 0.48 | 1.67 | 1.80 | 60 |
| No.160 | 1.80 | M | 64 | Yes | No | 112 | 60 | 3.29 | 4.47 | 2.68 | 2.98 | 0.90 | 55 |
| No.161 | 1.94 | F | 44 | No | No | 121 | 67 | 4.17 | 5.11 | 0.80 | 3.61 | 1.35 | 57 |
| No.162 | 2.09 | F | 67 | No | No | 130 | 80 | 6.61 | 1.81 | 2.32 | 4.62 | 2.12 | 37 |
| No.163 | 2.11 | M | 73 | No | No | 121 | 65 | 3.72 | 1.14 | 5.84 | 1.38 | 3.91 | 38 |
| No.164 | 2.04 | F | 81 | No | No | 125 | 80 | 4.30 | 0.91 | 5.67 | 1.56 | 3.70 | 40 |
| No.165 | 2.06 | M | 69 | No | No | 123 | 64 | 5.73 | 1.31 | 2.83 | 1.46 | 2.71 | 40 |
| No.166 | 1.98 | F | 66 | No | No | 112 | 65 | 6.46 | 1.68 | 2.58 | 1.41 | 1.49 | 40 |
| No.167 | 1.96 | M | 57 | No | No | 150 | 90 | 4.36 | 1.47 | 6.10 | 1.52 | 1.53 | 40 |
| No.168 | 2.04 | M | 66 | No | No | 162 | 90 | 5.21 | 1.10 | 3.90 | 1.26 | 2.52 | 40 |
| No.169 | 1.91 | M | 80 | No | No | 130 | 60 | 5.43 | 1.79 | 2.33 | 1.13 | 1.92 | 45 |
| No.170 | 2.02 | M | 66 | No | No | 125 | 70 | 5.62 | 1.27 | 2.47 | 1.37 | 3.61 | 45 |
| No.171 | 1.98 | M | 61 | No | No | 140 | 90 | 3.42 | 2.48 | 2.90 | 1.08 | 4.95 | 45 |
| No.172 | 1.92 | M | 64 | No | No | 137 | 84 | 3.93 | 1.52 | 1.90 | 1.41 | 1.04 | 46 |
| No.173 | 2.06 | M | 70 | No | No | 142 | 78 | 3.46 | 3.02 | 4.58 | 1.02 | 2.01 | 38 |
| No.174 | 1.97 | M | 66 | No | No | 140 | 80 | 4.85 | 1.06 | 1.32 | 1.41 | 3.86 | 45 |
| No.175 | 2.03 | M | 63 | Yes | No | 136 | 89 | 3.38 | 1.66 | 4.50 | 1.13 | 1.05 | 40 |
| No.176 | 1.93 | M | 60 | No | No | 140 | 85 | 4.21 | 2.18 | 1.55 | 1.13 | 3.92 | 45 |
| No.177 | 2.04 | M | 56 | Yes | Yes | 134 | 83 | 3.28 | 4.50 | 2.20 | 0.99 | 2.19 | 38 |
| No.178 | 2.06 | M | 48 | Yes | Yes | 169 | 90 | 3.42 | 1.29 | 3.45 | 1.52 | 2.50 | 38 |
| No.179 | 1.99 | M | 87 | No | No | 143 | 57 | 3.17 | 0.65 | 1.12 | 1.73 | 2.26 | 45 |
| No.180 | 1.88 | M | 82 | Yes | Yes | 106 | 54 | 4.63 | 3.71 | 2.89 | 1.09 | 1.59 | 48 |
| No.181 | 1.99 | M | 73 | No | No | 110 | 70 | 3.77 | 1.88 | 3.62 | 1.31 | 0.92 | 44 |
| No.182 | 1.94 | M | 73 | Yes | No | 150 | 70 | 3.00 | 0.77 | 3.04 | 0.95 | 1.84 | 45 |
| No.183 | 1.95 | M | 69 | No | No | 121 | 60 | 3.03 | 1.68 | 2.35 | 1.00 | 1.14 | 45 |
| No.184 | 1.89 | F | 81 | No | No | 130 | 60 | 3.11 | 1.60 | 1.97 | 2.64 | 1.54 | 48 |
| No.185 | 1.88 | M | 57 | No | No | 151 | 76 | 4.83 | 0.91 | 3.37 | 2.62 | 0.73 | 49 |
| No.186 | 1.87 | F | 60 | No | No | 150 | 90 | 3.30 | 2.16 | 1.27 | 2.60 | 1.37 | 48 |
| No.187 | 1.87 | F | 64 | No | No | 126 | 77 | 3.11 | 1.20 | 1.00 | 3.27 | 0.73 | 48 |
| No.188 | 1.86 | M | 58 | No | No | 130 | 80 | 4.84 | 1.51 | 1.62 | 3.05 | 1.13 | 49 |

Data collected included all participant information. No.1 to No.40, normal population; No.41 to No.74, type A dissection patients; No.75 to No.81, type B dissection aneurysm patients; No.82 to No.188, CAS patients. F, represents female; M, represents male; SBP, systolic blood pressure; DBP, diastolic blood pressure; FPG, fasting plasma glucose; TC, total cholesterol; TG, triglyceride; HDL-c, high-density lipoprotein cholesterol; LDL-c, low-density lipoprotein cholesterol.
